# Supplementary material for: Temporal-iCLIP captures co-transcriptional RNA-protein interactions
Source: Nat Commun. 2023 Feb 8;14:696. doi: 10.1038/s41467-023-36345-y (PMC9908952; doi:10.1038/s41467-023-36345-y)
Supplement: Supplementary file 1 — Supplementary Information [file 41467_2023_36345_MOESM1_ESM.pdf]

Supplementary Information

Temporal iCLIP captures co-transcriptional RNA-protein interactions

Ross A. Cordiner[1,2], Yuhui Dou[1,3], Rune Thomsen[1], Andrii Bugai[1], Sander Granneman[2], Torben Heick Jensen[1,*]

[1] Department of Molecular Biology and Genetics, Aarhus University, Universitetsbyen 81, 8000 Aarhus, Denmark

[2] Centre for Engineering Biology, University of Edinburgh, Mayfield Road, Edinburgh, EH9 3JD, UK

[3] Present address: Friedrich Miescher Institute for Biomedical Research, 4058 Basel, Switzerland

[*] Corresponding author: thj@mbg.au.dk

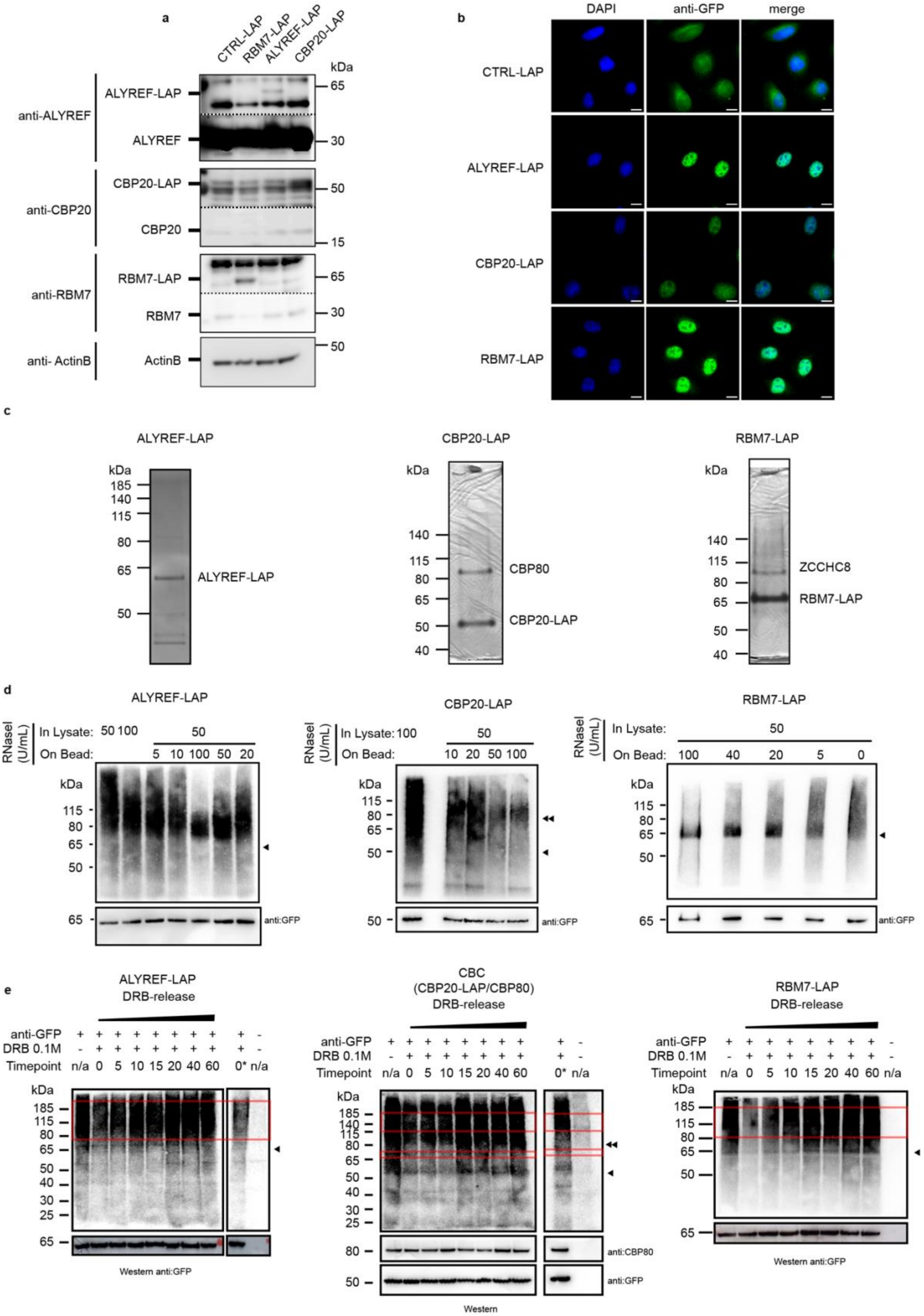
a
CTRL-LAP
RBM7-LAP
ALYREF-LAP
CBP20-LAP
kDa
anti-ALYREF
ALYREF-LAP
ALYREF
anti-CBP20
CBP20-LAP
CBP20
anti-RBM7
RBM7-LAP
RBM7
anti- ActinB
ActinB
65
30
50
15
65
30
50
b
DAPI
anti-GFP
merge
CTRL-LAP
ALYREF-LAP
CBP20-LAP
RBM7-LAP
c
ALYREF-LAP
CBP20-LAP
RBM7-LAP
kDa
185
140
115
80
65
50
ALYREF-LAP
CBP80
CBP20-LAP
ZCCHC8
RBM7-LAP
40
d
ALYREF-LAP
CBP20-LAP
RBM7-LAP
RNaseI (U/mL)
In Lysate: 50 100
50
On Bead: 5 10 100 50 20
In Lysate: 100
50
On Bead: 10 20 50 100
In Lysate:
50
On Bead: 100 40 20 5 0
anti:GFP
e
ALYREF-LAP
DRB-release
CBC
(CBP20-LAP/CBP80)
DRB-release
RBM7-LAP
DRB-release
anti-GFP
DRB 0.1M
Timepoint n/a 0 5 10 15 20 40 60 0* n/a
Western anti:GFP
anti:CBP80
anti:GFP
Western
25

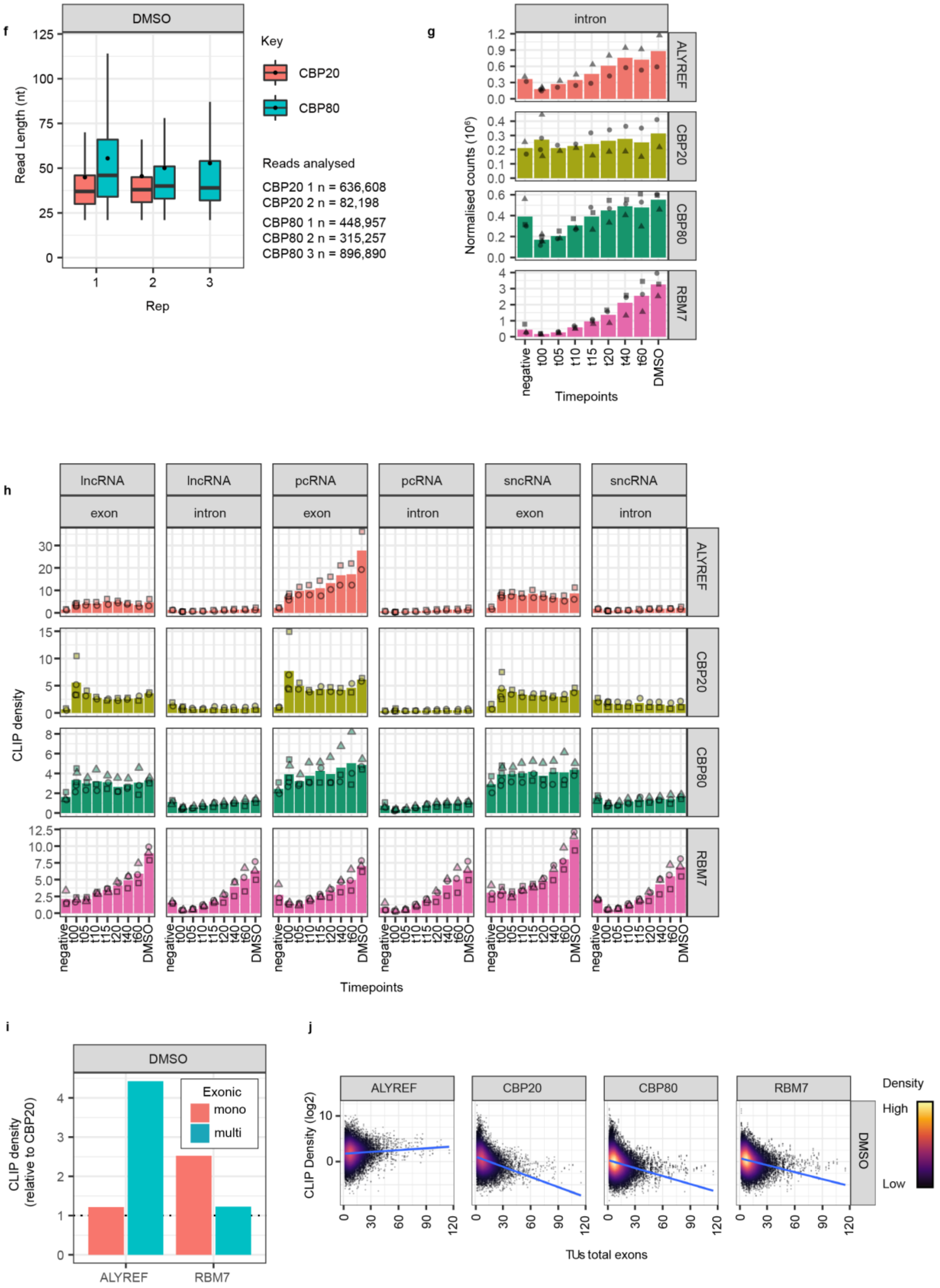

Supplementary Fig. 1. Temporal iCLIP uncovers dynamic RBP-RNA binding profiles (related to Fig. 1). a Western blotting analysis of LAP-tagged proteins and their un-tagged endogenous counterparts for all of the cell lines used. Panels separated by dashed horizontal lines are part of the same exposure, but have the intervening western blot cropped out. The control 'CTRL-LAP' cell line expressed the LAP-tag only. **b** Subcellular localisation analysis of the indicated LAP-tagged proteins. White horizontal scale bar represents 10μm. Displayed is a representative image of two independent

experiments. **c** SDS-PAGE followed by silver staining of immunoprecipitations of the LAP-tagged proteins. Note the co-purifying CBP80 (mid panel) and ZCCHC8 (right panel) proteins. **d** Autoradiograms displaying cross-linked and ^{32}P-labelled RNPs immunoprecipitated by the indicated LAP-tagged proteins treated with varying RNaseI concentration (see Methods). Black arrows indicate the molecular weights of LAP-tagged proteins (double arrows indicate endogenous CBP80). Equal loading was demonstrated by anti-GFP western blotting analysis shown in the bottom panels. Lack of radioactive signal associated with the CBP20-RNPs (middle panel, single black arrow) is likely explained by cross-linking of CBP20 close to the 5' cap; preventing radiolabelling as the alkaline phosphatase enzyme does not convert trimethyl caps into the necessary 5'OH substrates. **e** Autoradiograms displaying cross-linked and ^{32}P-labelled RNPs immunoprecipitated by the indicated LAP-tagged proteins and separated by SDS-PAGE. Note that a second timepoint 0 (0*; referred to as t00-2 in the genome browser views) was collected for the ALYREF and CBC samples. Blank magnetic beads were used as negative controls (negative anti-GFP lanes) on unsynchronised samples. Red boxes illustrate where membranes were cut to collect material for RNA sequencing and black arrows were used as in **d.** The same membranes were blotted with anti-GFP antibodies to confirm factor migration and equal loading (panel below all autoradiograms). The 'CBC membrane' was also probed with anti-CBP80 antibodies. **f** Boxplot displaying median (bands), first and third quartiles (box), ± 1.5 × interquartile range (whiskers) and mean (black circles) read lengths generated from CBP20- and CBP80-DMSO tiCLIP libraries. **g** Histograms as in Fig. 1d, but showing normalised read counts mapping to introns. 11120 TUs were used in this analysis. **h** Histograms as in Fig. 1d, but for exonic and intronic regions of transcripts categorised by their coding capacity and length (long non-coding RNA (lncRNA) >200nt; protein coding RNA (pcRNA) ; short noncoding RNA (sncRNA) <200nt). **i** Histograms displaying the relative binding density for multi- and mono-exonic transcripts normalised to the CBP20-DMSO sample. DMSO timepoints shown. **j** Scatter plots showing RBP tiCLIP read densities *vs.* TU exon content over a total of 11240 TUs. Blue line represents linear model of data. Heat scale represents density of data points. Source data are available in the Source Data file.

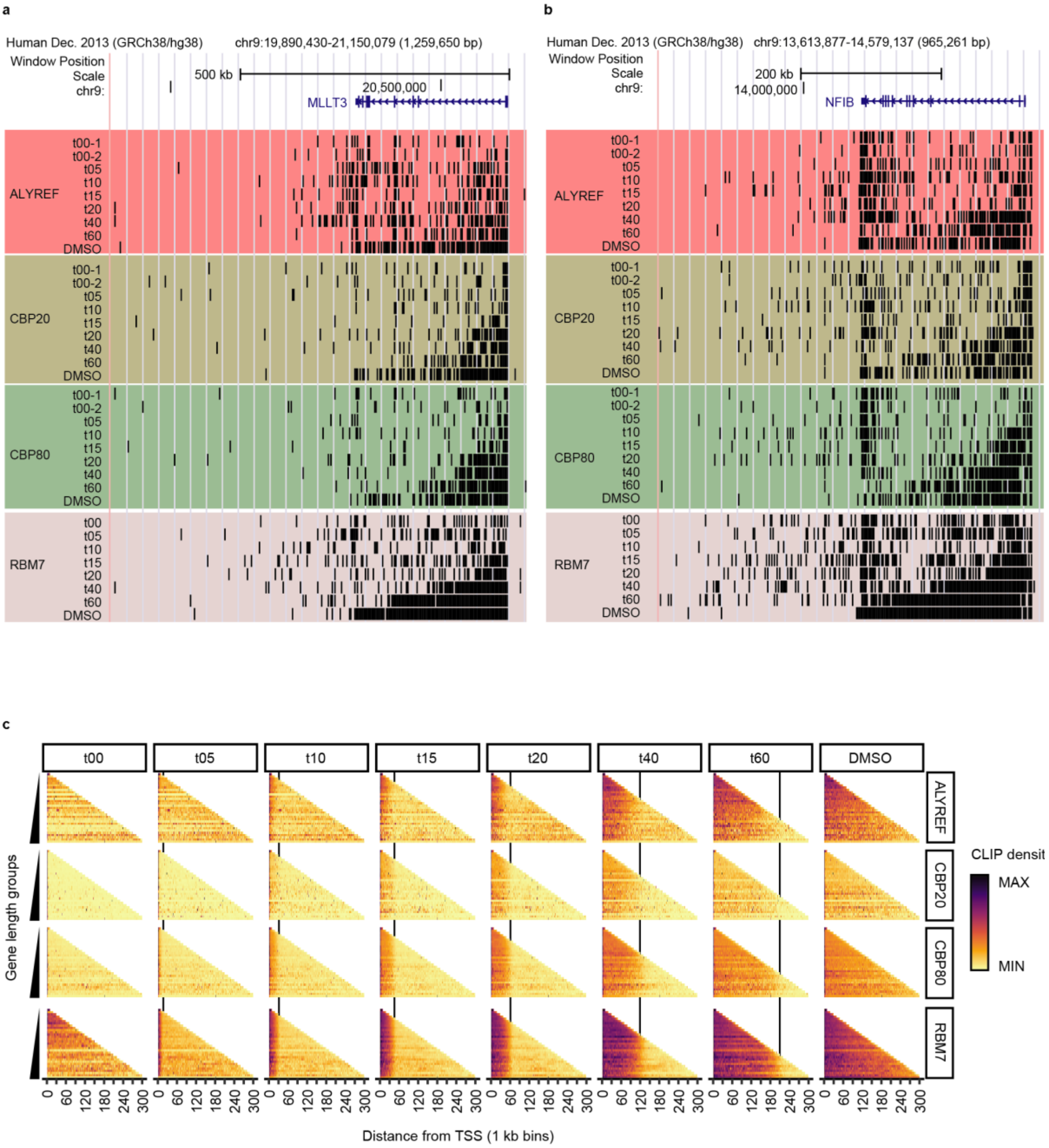

Supplementary Fig. 2. Spatiotemporal RNA binding of the CBC, ALYREF and RBM7 is dictated by RNAPII transcription (related to Fig. 2). a-b UCSC genome browser views of MLLT3 (**a**) and NFIB (**b**) loci, depicting regions with mapped ticLIP reads (in black) from the indicated IP samples and timepoints. Only reads mapping to the minus strand are shown. **c** Heatmaps as in Fig. 2a, but generated using intronic reads only. Source data are available in the Source Data file.

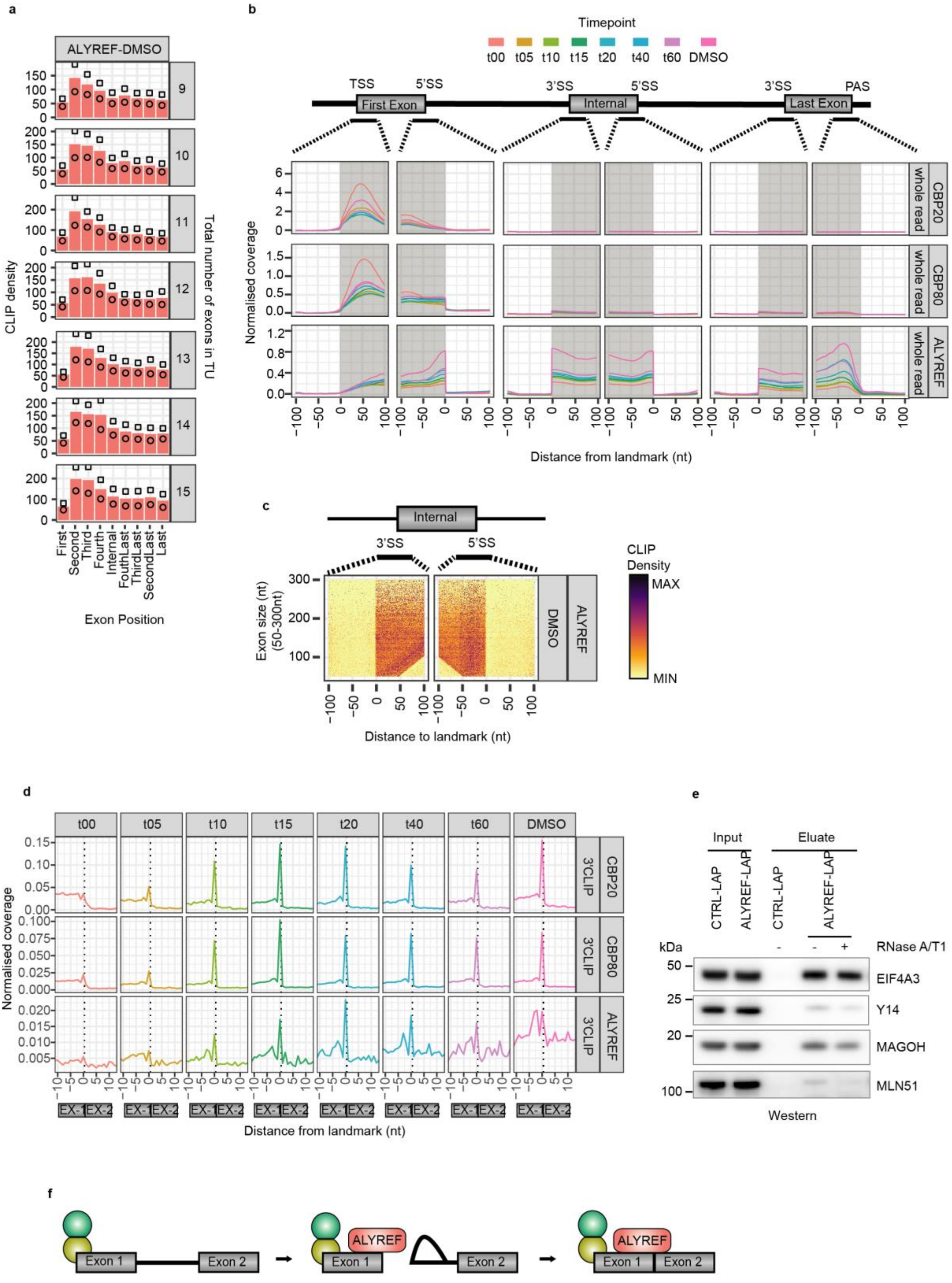

Supplementary Fig. 3. ALYREF and the CBC bind specific RNA splicing intermediates (related to Fig. 3). a Histogram as in Fig. 3b, but stratifying the steady-state data for ALYREF (DMSO) by the total number of exons per transcript as indicated to the right of the histograms. 5698 TUs used in this analysis. **b** Aggregate plots displaying normalised coverage as in Fig. 3d-f, but showing whole read signals. **c** Heatmaps showing ALYREF steady state (DMSO) read densities over a 201nt window centred around the 5' (left panel) or 3' (right panel) -ends of internal exons grouped by their lengths

(50nt – 300nt). The values for each exon size group were normalised to the max value within that group. **d** Aggregate plots displaying normalised 3'CLIP data coverage over the last 10nt of exon 1 (EX-1) and the first 10nt of exon 2 (EX-2) for CBP20 (top), CBP80 (middle) and ALYREF (bottom). Dotted vertical line identifies exon-exon junction. **e** Western blotting analysis of ALYREF-LAP immunoprecipitates, revealing RNA-independent interactions with core members of the EJC. Note that a panel probed with anti-GFP to display equal amounts of immunoprecipitated ALYREF-LAP was previously published as Figure 2D in Dou et. al 2020[1]. Displayed is a representative image of two independent experiments. **f** Schematic representation of the timing by which the CBC and ALYREF associate with the nascent RNA. Source data are available in the Source Data file.

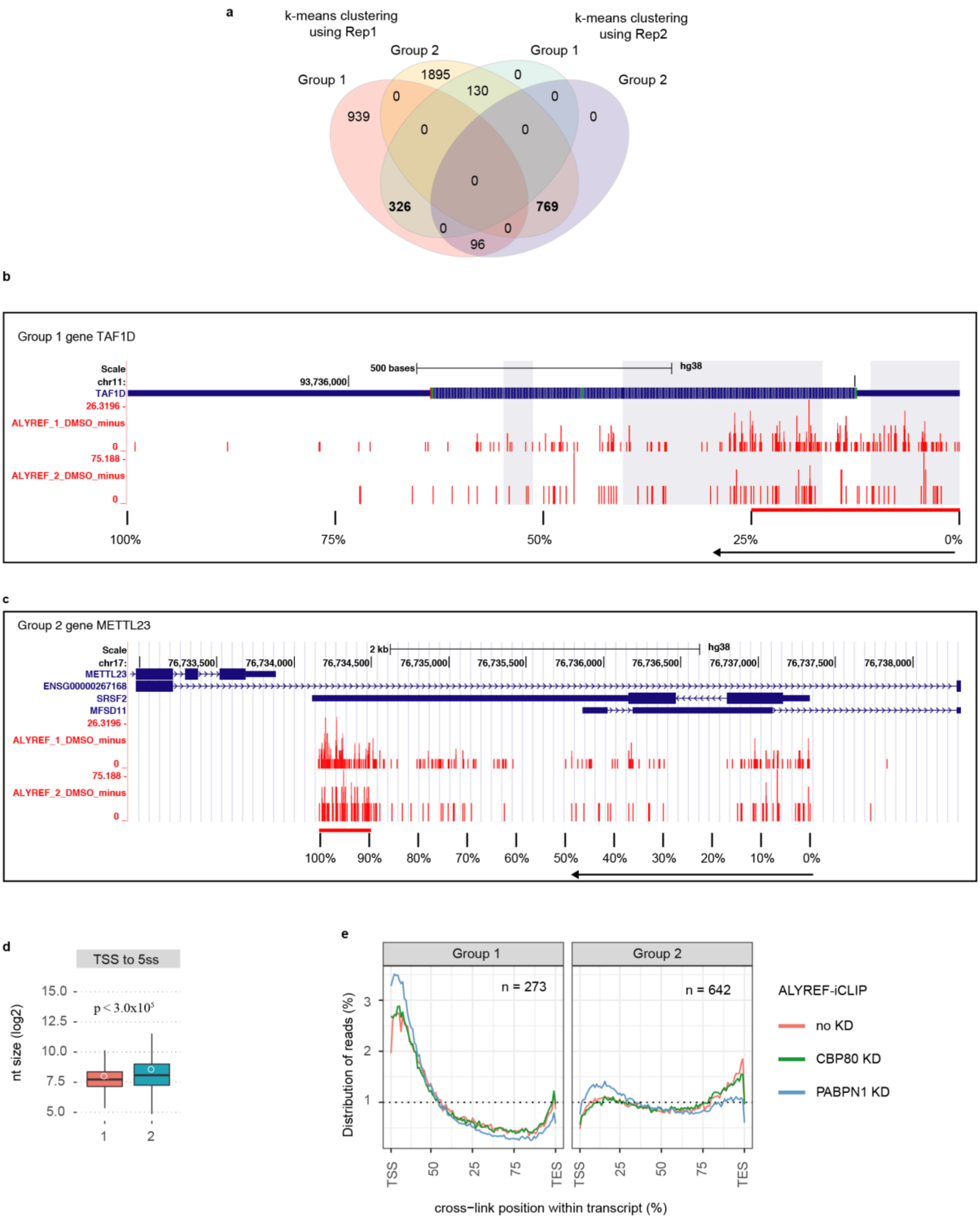

Supplementary Fig. 4. Transcript features dictate different ALYREF anchoring profiles (related to Fig. 4). a Intersection of transcription unit (TU) lists produced by k-means clustering of the independent ALYREF-DMSO replicate data (see Methods). **b-c** UCSC genome browser views of representative TUs from group 1 (**b**) or group 2 (**c**) as defined in **a**. Percentiles of TU lengths are indicated and red horizontal bars mark the first 25% (**b**) or last 10% (**c**) of the TUs. Arrows represent transcription directions. Only minus strand data are shown. Note, that for TAF1D (**b**) introns are collapsed and exons are marked by alternating white and blue shading. Red horizontal line mark high read density regions. **d** Boxplots showing distances from transcription start sites (TSSs) to the downstream 5'SSs of group 1 (red) and 2 (blue) TUs from Fig. 4b. A two-sided Wilcox rank sum test

was used to compare the means. No adjustments were made for multiple comparisons. p value is shown on the figure. Group 1 n = 326 genes assessed over 2 biological replicates; Group 2 n = 769 genes assessed over 2 biological replicates. **e** Average cross-link distribution profiles as in Fig. 4a, but generated from ALYREF CLIP data from Shi et al. 2017[2]. All TUs were normalised for length, and distribution displays reads that mapped to exonic RNA (mature RNA). Profiles were stratified into TU groups identified in **a**. The number of TUs used for analysis are displayed on the relative panel. Only TUs with CLIP signal in all ALYREF iCLIP conditions were used. 'No KD' represents a non-siRNA treated control. Source data are provided as a Source Data file.

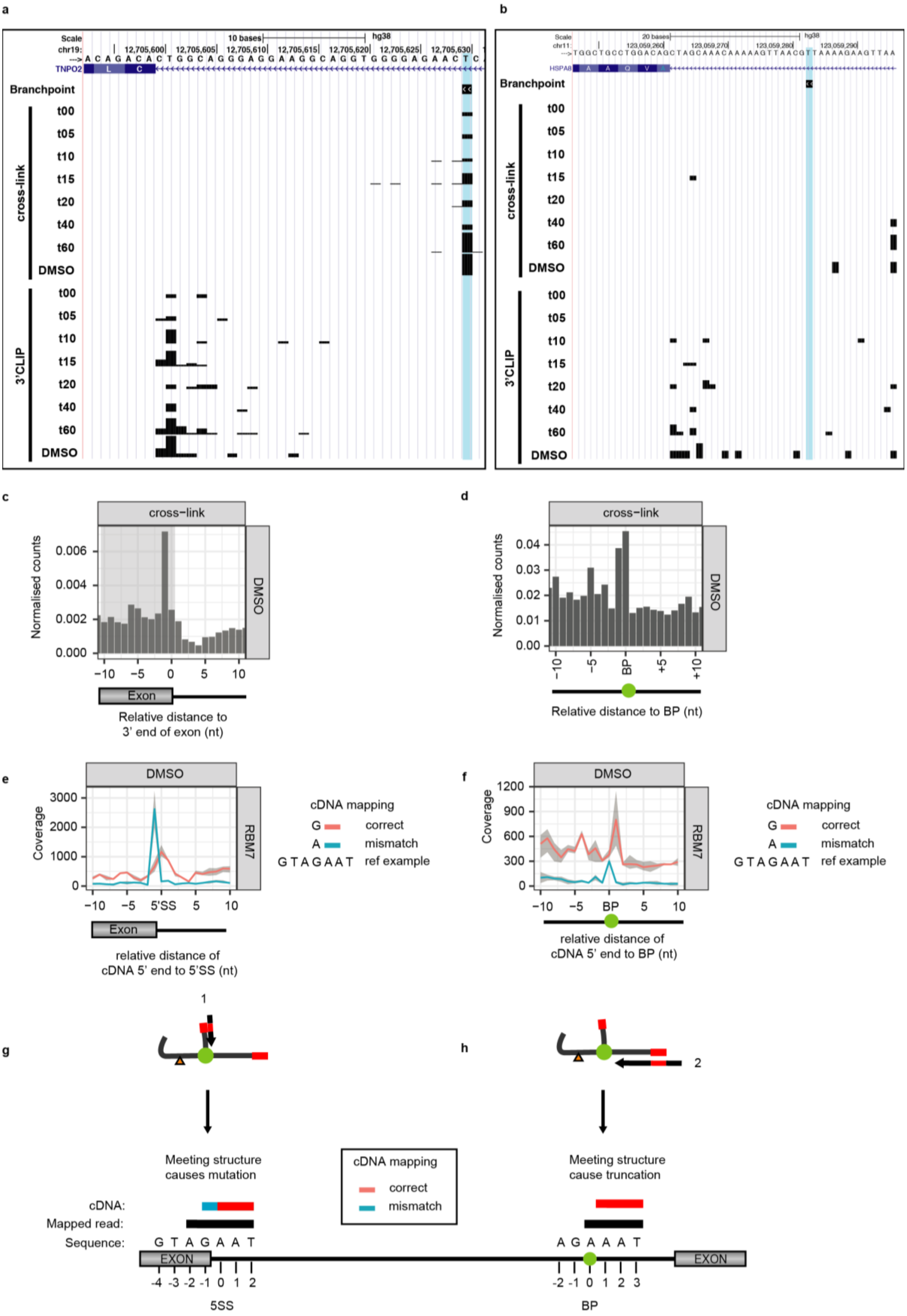

Supplementary Fig. 5. RBM7 is recruited to introns before debranching but after the second transesterification step (related to Fig. 5). a-b UCSC genome browser views displaying representative introns with BP to 3'SS distances 30nt (**a**) or 21nt (**b**). RBM7 tiCLIP cross-link and 3'CLIP

coverages are shown for the minus strand only. Cross-link and 3'CLIP tracks are group scaled, respectively. Blue shading identifies BP. **c**-**d** Quantification of RBM7 cross-link sites over exon-intron junctions (**c**) or BPs (**d**). Shaded area indicates exonic regions. **e** Aggregate plot of positions of the first nucleotide of CLIP reads relative to the 3'ends of exons. Colours indicate whether the 5'nts of reads matched the reference sequence (red) or not (blue). Grey ribbon represents confidence intervals of biological replicate samples. **f** Aggregate plot as in (**e**) but centred around BPs. **g**-**h** A schematic representation of cDNAs generated from the circularised (**g**) or linear (**h**) part of the lariat and their respective effects on cDNA production. '1' and '2' labelled RT primers are as in Fig. 5d. 6684 exon-intron junctions were analysed in **c** and **e**. 3782 BPs were analysed in **d** and **f**. Source data are available in the Source Data file.

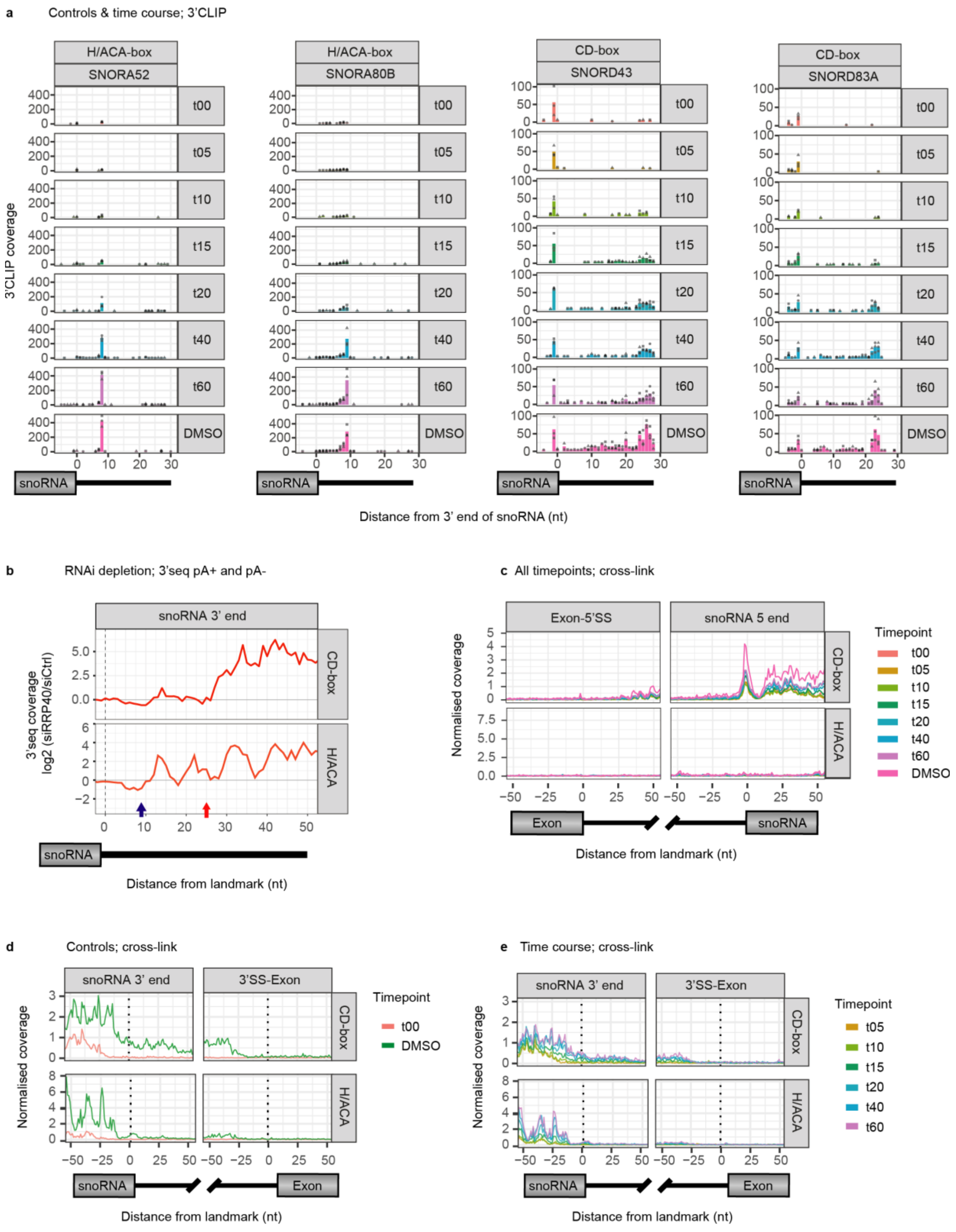

Supplementary Fig. 6. RBM7 binds specific snoRNA intermediates (related to Fig. 6). **a** 3'CLIP data coverage for RBM7 samples plotted at the 3'ends of representative CD- and H/ACA-box snoRNAs as indicated. Averages of 3 replicates are shown circles, squares and triangles represent individual replicates. **b** Coverage of RNA 3'end-seq pA^{+}/pA^{-} data from Wu et al. 2020[3] plotted downstream of CD (top)- or H/ACA (bottom)-box snoRNAs. Average data from 3 replicates were converted into log2 ratios of signals in siRRP40 *vs.* control samples. Red and blue arrows mark 3'extensions as displayed in Fig. 6g. **c** RBM7 cross-linking data plotted over a 100nt window anchored at snoRNA 5'ends (right panel) and the upstream 5'SSs (left panel) for all timepoints. Plot is stratified by CD (top)- and H/ACA (bottom-

box snoRNAs. **d-e** as in Fig. 6a-b, but plotting RBM7 cross-linking data. Source data are provided as a Source Data file.

Supplementary Table 1. Table displaying key reagents, antibodies and oligo sequences used in this study.

Misc.		
**Reagent**	**Product Line**	**Cat No.**
RNase I 100U/ul	Ambion	AM2295
0.2ml PCR Tube, Flat Caps, MaxyMum Recovery, Clear	Axygen	PCR-02-L-C
CircLigase™ II ssDNA Ligase	EpiCentre	CL9025K
DNA LOBIND TUBES 1,5ML PCR CLEAN	Eppendorf	525-0130
LoBind Tubes 2mL	Eppendorf	30108078
LoBind Tubes 1.5mL	Eppendorf	30108051
NuPAGE LDS Sample Buffer	Invitrogen	C-NP0007-X
SSIV	Invitrogen	8090050
RNASEOUT RECOM RIBONUC INHIBITOR 5,000 UNITS	Invitrogen	C-10777019-X
Novex TBE Urea gels 6% 10 well	Invitrogen	EC6865BOX
NuPAGE Novex 4-12% Bis-Tris Protein 1.0mm 10 well	Invitrogen	NP0321BOX
Accuprime supermix I	Invitrogen	12342010
Turbo DNase	Ambion	AM2238
T4 RNA Ligase 2 Truncated KQ	NEB	M0373L
T4 PNK	NEB	M0201L
5 DNA Adenylation Kit	NEB	E2610L
NuPAGE™ MOPS SDS Running Buffer (20X)	Invitrogen	NP0001
NuPAGE™ Transfer Buffer (20X)	Invitrogen	NP00061
NuPAGE™ LDS Sample Buffer (4X)	Invitrogen	NP0007
cOmplete(TM) ULTRA Tablets, Mini, EDTA-free, EASYpack Protease Inhibitor Cocktail Tablet	Roche	5892791001
PEG average Mn 400	Sigma Aldrich	202398-g
Costar Column	Sigma-Aldrich Merck	CLS8160-96EA
Whatman® glass microfiber filters, Grade GF/D	Sigma-Aldrich Merck	WHA1823010
FastAP Thermosensitive Alkaline Phosphatase (1 U/uL)	ThermoFisher Scientific	EF0651
**Antibodies**		
**Reagent**	**Supplier**	**Cat No.**
anti-GFP	Santa Cruz	sc-9996
anti-CBP80	Gift from the Elisa Izzauralde laboratory	N/A
anti-Y14	Gift from the Herve Le Hir laboratory	N/A
anti-MLN51	Gift from C.L. Tomasetto	N/A
anti-EIF4A3	Gift from the Herve Le Hir laboratory	N/A
anti-MAGOH	Santa Cruz	sc56724
GFP-TRAP_MA	Chromotek	gtma-20
Binding Control MA	Chromotek	btma-20
anti-ALYREF	Abcam	ab202894
anti-RBM7	ProteinTech	21896-1-AP
goat anti-rabbit-hrp polyclonal antibody	Dako	P0448
goat anti-mouse-hrp polyclonal antibody	Dako	P0447
Alexa 488 conjugated Goat Anti mouse IgG (H+L)	ThermoFisher	A-11001
**Oligos**		
**ID**	**Sequence**	**Purification Method**
L3_pDNA_BC03	/5Phos/NTTTCTAACAGATCGGAAGAGCGGTTCAG/3SpC3/	Rnase Free HPLC
L3_pDNA_BC08	/5Phos/NTACAGATGAGATCGGAAGAGCGGTTCAG/3SpC3/	Rnase Free HPLC
L3_pDNA_BC15	/5Phos/NACATTATTAGATCGGAAGAGCGGTTCAG/3SpC3/	Rnase Free HPLC
L3_pDNA_BC17	/5Phos/NTACAACATAGATCGGAAGAGCGGTTCAG/3SpC3/	Rnase Free HPLC
L3_pDNA_BC18	/5Phos/NGCAGCCACAGATCGGAAGAGCGGTTCAG/3SpC3/	Rnase Free HPLC
L3_pDNA_BC21	/5Phos/NCGGAGGGCAGATCGGAAGAGCGGTTCAG/3SpC3/	Rnase Free HPLC
L3_pDNA_BC24	/5Phos/NATCACTTGAGATCGGAAGAGCGGTTCAG/3SpC3/	Rnase Free HPLC
L3_pDNA_BC25	/5Phos/NAGAATTATAGATCGGAAGAGCGGTTCAG/3SpC3/	Rnase Free HPLC
L3_pDNA_BC26	/5Phos/NGGCCCAAGAGATCGGAAGAGCGGTTCAG/3SpC3/	Rnase Free HPLC
L3_pDNA_BC31	/5Phos/NAAGTGTTGAGATCGGAAGAGCGGTTCAG/3SpC3/	Rnase Free HPLC
cut_Rt1clip	/5Phos/NNAACCNNNAGATCGGAAGAGCGTCGTGgatcCTGAACCGC	Standard Desalting
cut_Rt2clip	/5Phos/NNACAANNNAGATCGGAAGAGCGTCGTGgatcCTGAACCGC	Standard Desalting
cut_Rt3clip	/5Phos/NNATTGNNNAGATCGGAAGAGCGTCGTGgatcCTGAACCGC	Standard Desalting
cut_Rt4clip	/5Phos/NNAGGTNNNAGATCGGAAGAGCGTCGTGgatcCTGAACCGC	Standard Desalting
cut_Rt6clip	/5Phos/NNCCGGNNNAGATCGGAAGAGCGTCGTGgatcCTGAACCGC	Standard Desalting
cut_Rt7clip	/5Phos/NNCTAANNNAGATCGGAAGAGCGTCGTGgatcCTGAACCGC	Standard Desalting

cut_Rt8clip	/5Phos/NNCATTNNNAGATCGGAAGAGCGTCGTGgatcCTGAACCGC	Standard Desalting
cut_Rt9clip	/5Phos/NNGCCANNNAGATCGGAAGAGCGTCGTGgatcCTGAACCGC	Standard Desalting
cut_Rt11clip	/5Phos/NNGGTTNNNAGATCGGAAGAGCGTCGTGgatcCTGAACCGC	Standard Desalting
cut_Rt12clip	/5Phos/NNGTGGNNNAGATCGGAAGAGCGTCGTGgatcCTGAACCGC	Standard Desalting
cut_Rt13clip	/5Phos/NNTCCGNNNAGATCGGAAGAGCGTCGTGgatcCTGAACCGC	Standard Desalting
cut_Rt14clip	/5Phos/NNTGCCNNNAGATCGGAAGAGCGTCGTGgatcCTGAACCGC	Standard Desalting
cut_Rt15clip	/5Phos/NNTATTNNNAGATCGGAAGAGCGTCGTGgatcCTGAACCGC	Standard Desalting
cut_Rt16clip	/5Phos/NNTTAANNNAGATCGGAAGAGCGTCGTGgatcCTGAACCGC	Standard Desalting
cut_oligo	GTTCAGGATCCACGACGCTCTTCAAAA	Standard Desalting
P5Solexa	AATGATACGGCGACCACCGAGATCTACACTCTTTCCCTACACGACGCTCTTCCGATCT	Standard Desalting
P3Solexa	CAAGCAGAAGACGGCATACGAGATCGGTCTCGGCATTCCTGCTGAACCGCTCTTCCGATCT	Standard Desalting

Supplementary Table 2. Composition of buffers used in the tiCLIP protocol.

**LB - Lysis Buffer**	**Working Conc.**
Tris/HCl, pH 8.0	50mM
NaCl	100mM
Triton-X100	0.5% (v/v)
Na-deoxycholate	0.25% (w/v)
EDTA	5mM
**To be added to 50mL aliquot fresh**	
1mM DTT (fresh)	1mM
complete EDTA-free protease inhibitor cocktail (Roche)	1x
SDS	0.10%
**HSW - High-salt wash buffer**	**Working Conc.**
Tris/HCl, pH 8.0	20mM
NaCl	1M
Triton-X100	0.5% (v/v)
Na-deoxycholate	0.5% (w/v)
EDTA	5mM
SDS	0.10%
**To be added to 50mL aliquot fresh**	
Urea (add 6g solid)	2M
DTT (add fresh)	1mM
complete EDTA-free protease inhibitor cocktail (Roche)	1x
**LCW - LiCl wash**	**Working Conc.**
Tris/HCl, pH 8.0	50mM
LiCl	250mM
TritonX100	0.5% (v/v)
Na-deoxycholate	0.5% (w/v)
EDTA	1mM
**NSB - No-salt wash buffer**	**Working Conc.**
Tris/HCl, pH 8.0	50mM
(v/v) Tween	0.2%
MgCl2	10mM
**DPB - Dephosphorylation buffer**	**Working Conc.**
Tris-HCl, pH 8.0	50mM
NaCl	100mM
MgCl2	10mM
**PWB - Phosphatase wash buffer**	**Working Conc.**
Tris-HCl, pH 7.5	50mM
EGTA (pH 8)	20mM
TritonX	0.5% (v/v)
**PNKW - PNK buffer**	**Working Conc.**
Tris-HCl, pH 7.5	50mM
NaCl	50mM
MgCl2	10mM
**LIGBx1 - Ligation Buffer (1x)**	**Working Conc.**
Tris-HCl	50mM
MgCl2	10mM

Supplementary References

1. Dou, Y. *et al.* NCBP3 positively impacts mRNA biogenesis. *Nucleic Acids Res.* **48**, 10413–10427 (2020) doi: 10.1093/nar/gkaa744.
2. Shi, M. *et al.* ALYREF mainly binds to the 5′ and the 3′ regions of the mRNA in vivo. *Nucleic Acids Res.* **45**, 9640–9653 (2017) doi: 10.1093/nar/gkx597.
3. Wu, G. *et al.* A Two-Layered Targeting Mechanism Underlies Nuclear RNA Sorting by the Human Exosome. *Cell Rep.* **30**, 2387-2401.e5 (2020) doi: 10.1016/j.celrep.2020.01.068.
