# Supplementary material for: Temporal-iCLIP captures co-transcriptional RNA-protein interactions
Source: Nat Commun. 2023 Feb 8;14:696. doi: 10.1038/s41467-023-36345-y (PMC9908952; doi:10.1038/s41467-023-36345-y)
Supplement: Supplementary file 3 — Description of Additional Supplementary Files [file 41467_2023_36345_MOESM3_ESM.pdf]

Description of Additional Supplementary Files

File Name: Supplementary Data 1
Description: Data displaying tiCLIP sample information; associated in-line rt and L3 barcode sequences and identifiers; mapped reads pre- and post-quality control, rRNA factor and normalised total mapped reads. Note CBP20, rep 3 (CBP20-3) was omitted from analysis due to a consistently low number of mapped reads across all timepoints and samples (under 20000; highlighted in red)

File Name: Supplementary Data 2
Description: TUs and corresponding cluster identities and attributes, identified from k-means clustering analysis of ALYREF-DMSO CLIP.
