# Supplementary material for: Temporal-iCLIP captures co-transcriptional RNA-protein interactions
Source: Nat Commun. 2023 Feb 8;14:696. doi: 10.1038/s41467-023-36345-y (PMC9908952; doi:10.1038/s41467-023-36345-y)
Supplement: Supplementary file 6 — Reporting Summary [file 41467_2023_36345_MOESM6_ESM.pdf]

nature portfolio

Corresponding author(s): Torben Heick Jensen
Last updated by author(s): 15/01/2023

Reporting Summary

Nature Portfolio wishes to improve the reproducibility of the work that we publish. This form provides structure for consistency and transparency in reporting. For further information on Nature Portfolio policies, see our Editorial Policies and the Editorial Policy Checklist.

Statistics

For all statistical analyses, confirm that the following items are present in the figure legend, table legend, main text, or Methods section.

n/a	Confirmed	
☐	☒	The exact sample size (*n*) for each experimental group/condition, given as a discrete number and unit of measurement
☒	☐	A statement on whether measurements were taken from distinct samples or whether the same sample was measured repeatedly
☐	☒	The statistical test(s) used AND whether they are one- or two-sided<br>*Only common tests should be described solely by name; describe more complex techniques in the Methods section.*
☒	☐	A description of all covariates tested
☐	☒	A description of any assumptions or corrections, such as tests of normality and adjustment for multiple comparisons
☐	☒	A full description of the statistical parameters including central tendency (e.g. means) or other basic estimates (e.g. regression coefficient) AND variation (e.g. standard deviation) or associated estimates of uncertainty (e.g. confidence intervals)
☐	☒	For null hypothesis testing, the test statistic (e.g. *F*, *t*, *r*) with confidence intervals, effect sizes, degrees of freedom and *P* value noted<br>*Give P values as exact values whenever suitable.*
☒	☐	For Bayesian analysis, information on the choice of priors and Markov chain Monte Carlo settings
☒	☐	For hierarchical and complex designs, identification of the appropriate level for tests and full reporting of outcomes
☒	☐	Estimates of effect sizes (e.g. Cohen's *d*, Pearson's *r*), indicating how they were calculated

Our web collection on statistics for biologists contains articles on many of the points above.

Software and code

Policy information about availability of computer code

Data collection	High-throughput sequencing: Illumina NextSeq550<br>Phosphoimaging: Typhoon FLA 9500 with control software v 1.1.0<br>Western Blot Imaging: Amersham ImageQuant 800 with ImageQuant 800 Control software v. 1.2.0
Data analysis	Demultiplexing and quality control<br>yCRAC software package (version 1.3.2) & pyBarcodeFilter.py (version 3.0).<br>umi_tools (version 0.5.5)<br>trim_galore (version 0.5.0)<br><br>Mapping and QC<br>hisat2 (version 2.1.0)<br>samtools (version 1.9)<br>umi_tools tool dedup f(version 0.5.5).<br><br>Making read1 and read2, and derivative cross-link and 3CLIP bigwig files<br>samtools (version 1.9)<br>bedtools (version v2.28.0)

bedGraphToBigWig (version 4) from UCSC tools.

Making annotation files
Bedtools (version 4)

Performing read counts across regions of interest
Bedtools (version 4)
dplyr (version 1.0.10)
tidyr (version 1.2.1)
ggplot2 (version 3.3.5).

k-means clustering analysis of ALYREF CLIP coverage profiles
stats (version 3.6.2)

Spatiotemporal RNA binding heatmaps
Bedtools (version 4)
dplyr (version 1.0.10)
tidyr (version 1.2.1)
ggplot2 (version 3.3.5).

RNA 3'seq pA+/pA- data analysis
Bedtools (version 4)
dplyr (version 1.0.10)
tidyr (version 1.2.1)
ggplot2 (version 3.3.5)

CBP20 and CBP80 read length analysis
dplyr (version 1.0.10)
tidyr (version 1.2.1)
ggplot2 (version 3.3.5).

The code used for data analysis can be found at https://github.com/racrna1989/tiCLIP_analysis.git.

For manuscripts utilizing custom algorithms or software that are central to the research but not yet described in published literature, software must be made available to editors and reviewers. We strongly encourage code deposition in a community repository (e.g. GitHub). See the Nature Portfolio guidelines for submitting code & software for further information.

Data

Policy information about availability of data

All manuscripts must include a data availability statement. This statement should provide the following information, where applicable:

- Accession codes, unique identifiers, or web links for publicly available datasets
- A description of any restrictions on data availability
- For clinical datasets or third party data, please ensure that the statement adheres to our policy

tiCLIP datasets GSE202980 [https://www.ncbi.nlm.nih.gov/geo/query/acc.cgi?&acc=GSE202980].
RNA 3'seq data GSE137612 [https://www.ncbi.nlm.nih.gov/geo/query/acc.cgi?acc=GSE137612].
ALYREF-CLIP data GSE99069 [https://www.ncbi.nlm.nih.gov/geo/query/acc.cgi?acc=GSE99069].
Source data for each figure are provided within the paper as a separate excel file.

Human research participants

Policy information about studies involving human research participants and Sex and Gender in Research.

Reporting on sex and gender	N/A
Population characteristics	N/A
Recruitment	N/A
Ethics oversight	N/A

Note that full information on the approval of the study protocol must also be provided in the manuscript.

Field-specific reporting

Please select the one below that is the best fit for your research. If you are not sure, read the appropriate sections before making your selection.

- [x] Life sciences
- [ ] Behavioural & social sciences
- [ ] Ecological, evolutionary & environmental sciences

For a reference copy of the document with all sections, see nature.com/documents/nr-reporting-summary-flat.pdf

Life sciences study design

All studies must disclose on these points even when the disclosure is negative.

Sample size	Sample sizes for CLIP, immunofluorescence and immunoblots were determined by financial and time limitations. Please not that ALYREF, CBP20 and CBP80 have two 0 min timepoint. We thought after processing the initial RBM7 tiCLIP library that it might be necessary to use 2 0 min timepoints to generate enough sequencing data for analysis.
Data exclusions	Biological replicate 3 of CBP20 was excluded from downstream analyses due to the low number of mapped reads across all timepoints (under 20,000). This exclusion is detailed in Supplementary table 1 which displays total mapped reads of all samples
Replication	2-3 biological replicates were processed for each tiCLIP sample. Each biological replicate was collected from different passage numbers. Where appropriate biological replicates were analysed independently and displayed as independent points on graphs, with the average of all the biological replicates displayed as a bar. Each replication was sucessful, except CBP20-3 mentioned above.<br><br>All other experiments were performed at least twice, on different days to ensure replication.
Randomization	Randomization for barcodes used in the tiCLIP protocol: The 10 different L3 barcoded adapters used for the 1st RNA ligation step in the iCLIP protocol were randomly allocated to different time points across the 11 tiCLIP experiments conducted to prevent barcode mediated sequencing biases.<br><br>For tiCLIP, immunofluorescence and immunoprecpitations experiments no randomization was required because millions of isogenic cells were used.
Blinding	Blinding was not required in this study because experiments were immunoprecpitations, immunofluorescence or tiCLIP experiments from common cell lines

Reporting for specific materials, systems and methods

We require information from authors about some types of materials, experimental systems and methods used in many studies. Here, indicate whether each material, system or method listed is relevant to your study. If you are not sure if a list item applies to your research, read the appropriate section before selecting a response.

Materials & experimental systems

n/a	Involved in the study	
☐	☒	Antibodies
☐	☒	Eukaryotic cell lines
☒	☐	Palaeontology and archaeology
☒	☐	Animals and other organisms
☒	☐	Clinical data
☒	☐	Dual use research of concern

Methods

n/a	Involved in the study	
☒	☐	ChIP-seq
☒	☐	Flow cytometry
☒	☐	MRI-based neuroimaging

Antibodies

Antibodies used	anti-GFP Santa Cruz sc-9996; clone: B-2; dilution: 1/1000 for western, 1/500 for immunofluorescence<br>anti-CBP80; gift from the Elisa Izzauralde laboratory (not commercially available) ; dilution 1/1000<br>anti-MLN51; gift from the Catherine Tomasetto laboratory (not commercially available); 1/1000<br>anti-Y14; gift from the Herve Le Hir laboratory (not commercially available); 1/500<br>anti-MAGOH Santa Cruz sc56724; 1/500<br>anti-EIF4A3; gift from the Herve Le Hir laboratory (not commercially available); 1/1000<br>GFP-TRAP_MA Chromotek gtma-20; amount used: 100uL per IP<br>Binding Control MA Chromotek btma-20; amount used: 100uL per IP

Alexa 488 conjugated Goat Anti mouse IgG (H+L) ThermoFisher A-11001 ; dilution 1/1000
goat anti-rabbit-hrp polyclonal antibody Dako P0448; dilution 1/10000
goat anti-mouse-hrp polyclonal antibody from Dako P0447; dilution 1/10000

Validation

anti-GFP (Santa Cruz sc-9996; clone: B-2) ; Immunoblot signal at expected size observed for every LAP-tagged protein used in this study immunoprecipitated from whole cell lysates generated from respective LAP-cell lines (Sup. Fig. . SantaCruz sc-9996 is cited in 2963 papers to date (01-DEC-2022).

anti-CBP80 (gift from the Elisa Izzauralde laboratory; not commercially available) ; immunoblot signal at expected size observed for endogenous CBP80 in CBP20-LAP precpitates.

anti-MLN51 ;immunoblot signal at expected size observed for endogenous MLN51 in HeLa whole cell lysates; usage also published in Degot et al. 2002 [https://doi.org/10.1038/sj.onc.1205611]

anti-Y14 ;immunoblot signal at expected size observed for endogenous Y14 in HeLa whole cell lysates

anti-MAGOH ;immunoblot signal at expected size observed for endogenous MAGOH in HeLa whole cell lysates

anti-EIF4A3 ;immunoblot signal at expected size observed for endogenous EIF4A3 in HeLa whole cell lysates

GFP-TRAP_MA Chromotek gtma-20 – Silver staining at expected size observed for SDS-PAGE analaysis of immunoprecipitation of LAP-tagged proteins with GFP-TRAP_MA beads. See Chromotek's validation statement below *

Binding Control MA Chromotek btma-20 No Silver staining observed for SDS-PAGE analysis of immunoprecipitations using Binding Control MA beads. See Chromotek's validation statement below *

*Validation statement from Chromotek's website (https://www.ptglab.com/products/chromotek-nanobody-based-reagents/about/about-nanobodies/?redirect=chromotek):

"ChromoTek utilizes genetic strategies and comparison with independent antibodies for the validation of our VHHs against fluorescent proteins and peptide tags:

In the genetic approach, VHHs are tested in their target application (immunoprecipitation, immunofluorescence and/or Western blot) both on cell lines that express and do not express their cognate fluorescent protein or peptide tag.
In addition, our Nanobodies are benchmarked with established conventional antibodies.
Our Nanobodies are always sequenced; in several cases, we even know their crystal structure. Furthermore, we thoroughly characterize and validate our monoclonal Nanobodies. Their recombinant production in combination with high QC standards ensures reliable and stable alpaca single domain antibody products virtually without lot-to-lot variations."

Eukaryotic cell lines

Policy information about cell lines and Sex and Gender in Research

Cell line source(s)

HeLa cells were obtained from ATCC.

HeLa Kyoto cells expressing GFP-tagged proteins were sourced from the Anthony Hyman Laboratory (poser et al. 2008 https://doi.org/10.1038/nmeth0808-748b).

Authentication

HeLa Kyoto cell lines were sequenced in their host lab for correct integration of the GFP encoding protein up or downstream of the relevant RNA binding proteins reading frame. Upon receiving the cells lines for ALYREF, RBM7 and CBP20, these cells were subjected to anti-GFP IPs and precipitates were assessed by SDS PAGE western blotting to confirm correct migration of GFP-tagged proteins (endogenous kDa + ~30kDa for GFP) and co-precipitation of previously defined interaction partners.

For IF and for ALYREF co-precipitates analysis, HeLa cells were used. HeLa cells morphology and behavior matched expectations.

Mycoplasma contamination

Cells lines tested negative for Mycoplasma contamination

Commonly misidentified lines
(See ICLAC register)

No commonly misidentified cell lines were used in this study.
