# Supplementary material for: Temporal-iCLIP captures co-transcriptional RNA-protein interactions
Source: Nat Commun. 2023 Feb 8;14:696. doi: 10.1038/s41467-023-36345-y (PMC9908952; doi:10.1038/s41467-023-36345-y)

Supplementary Fig. 1a

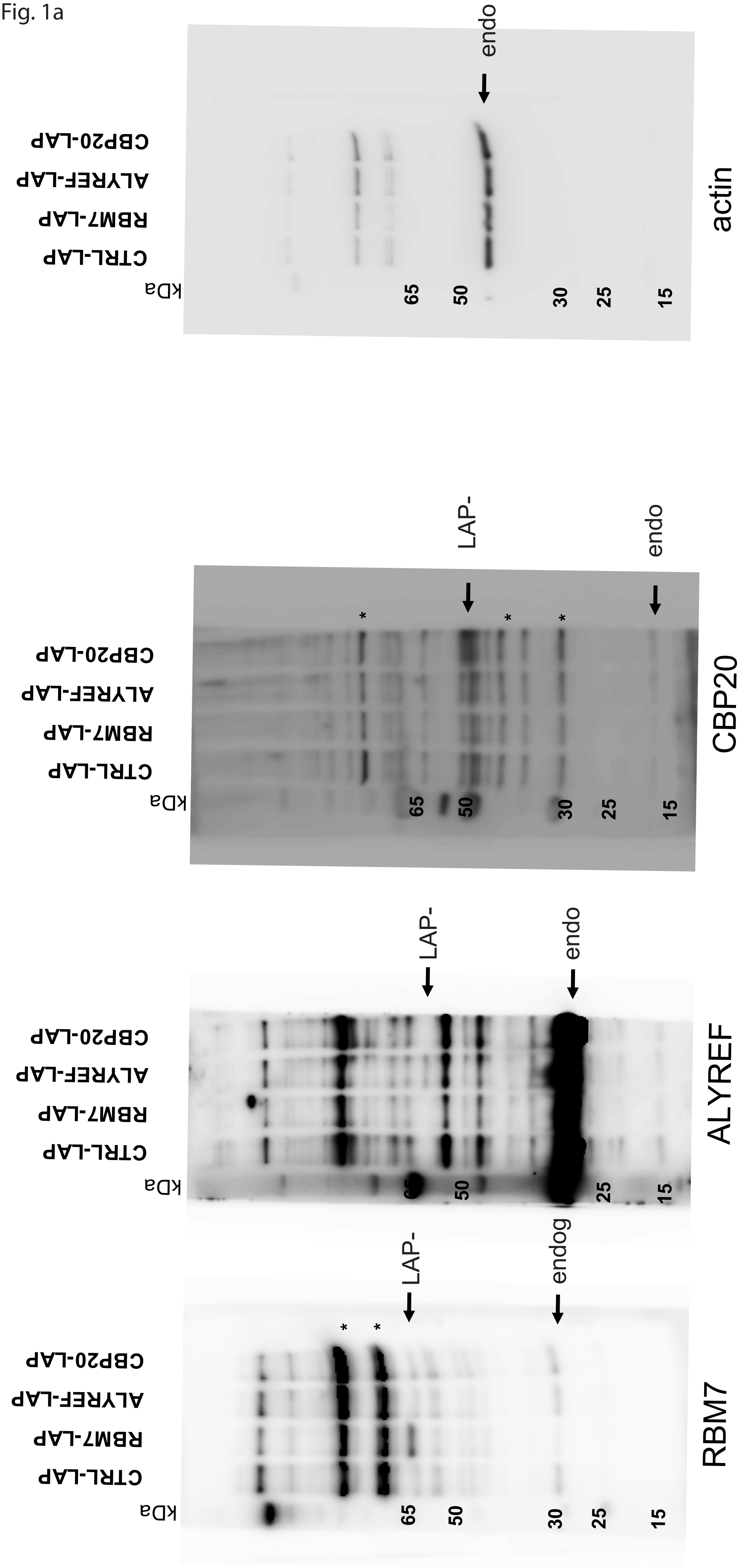

Supplementary Fig. 1b

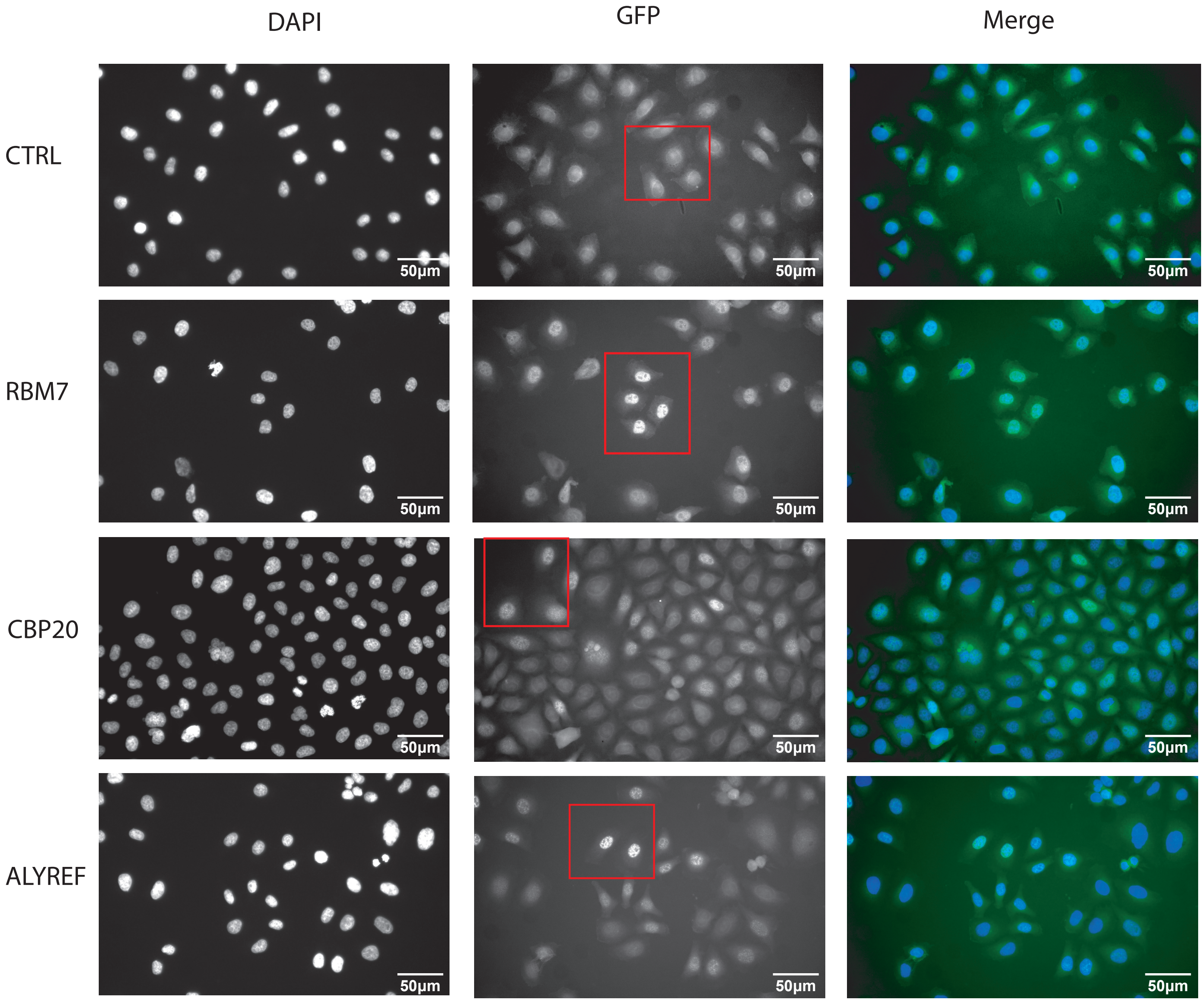

Red box represents region shown in figure

Supplementary Fig. 1c

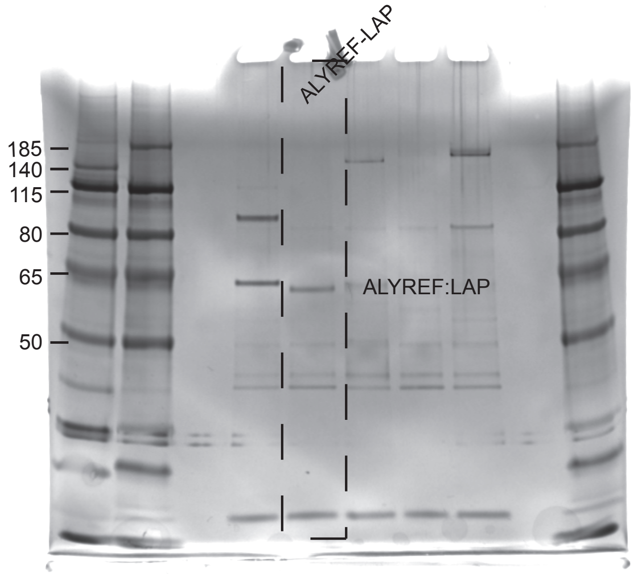

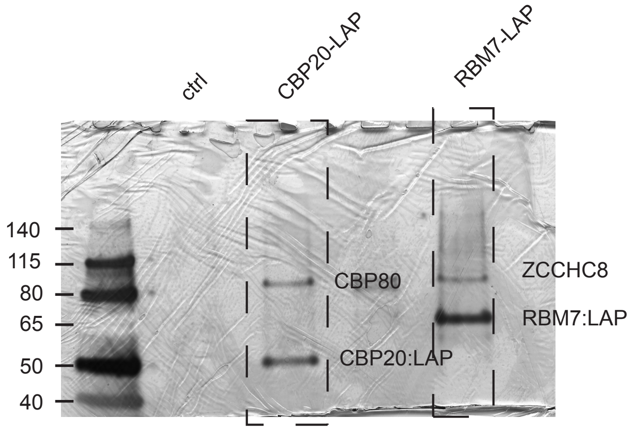

Dashed box represents region shown in figure

Supplementary Fig. 1d

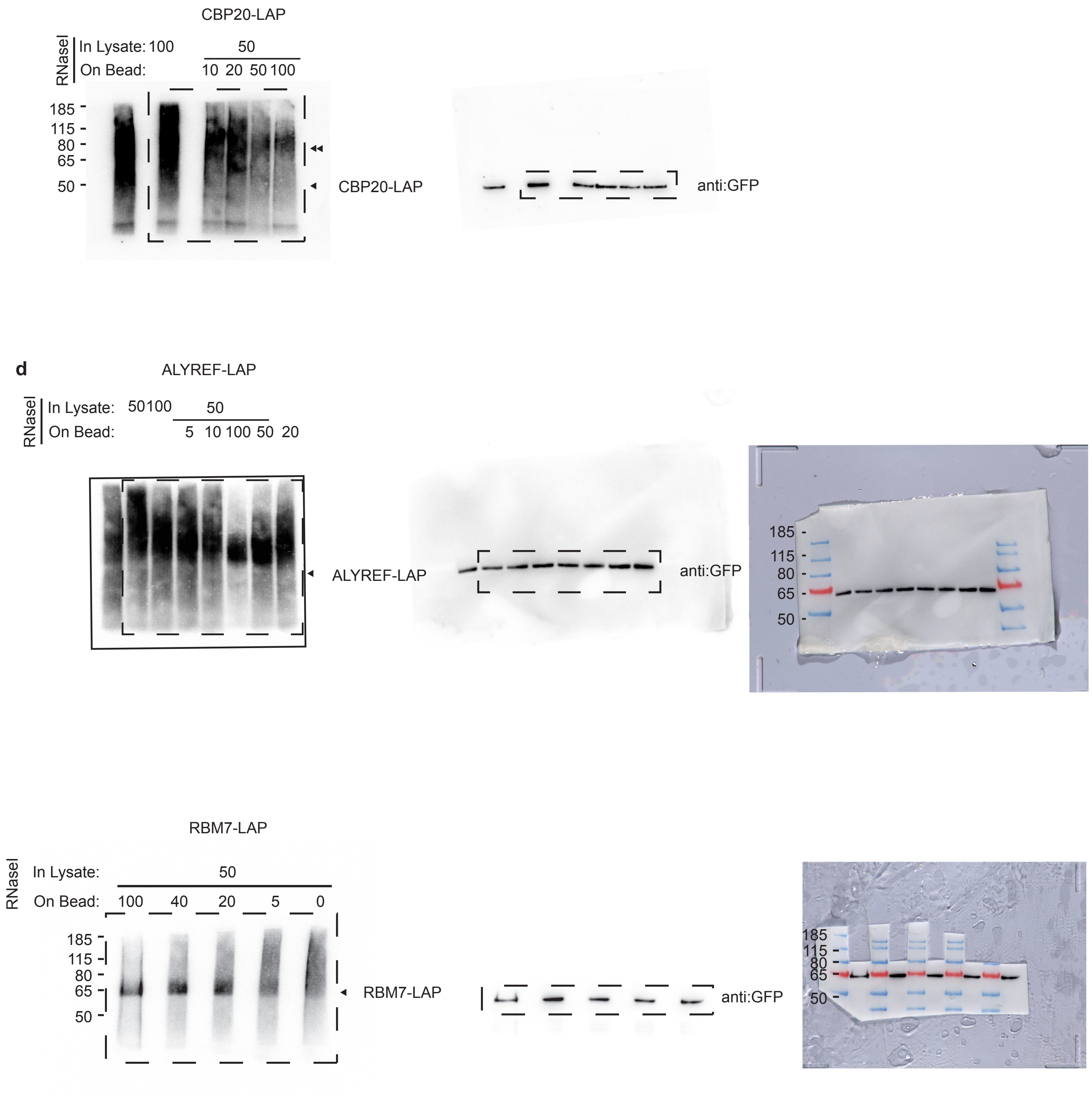

Dashed box represents region shown in figure

Supplementary Fig. 1e

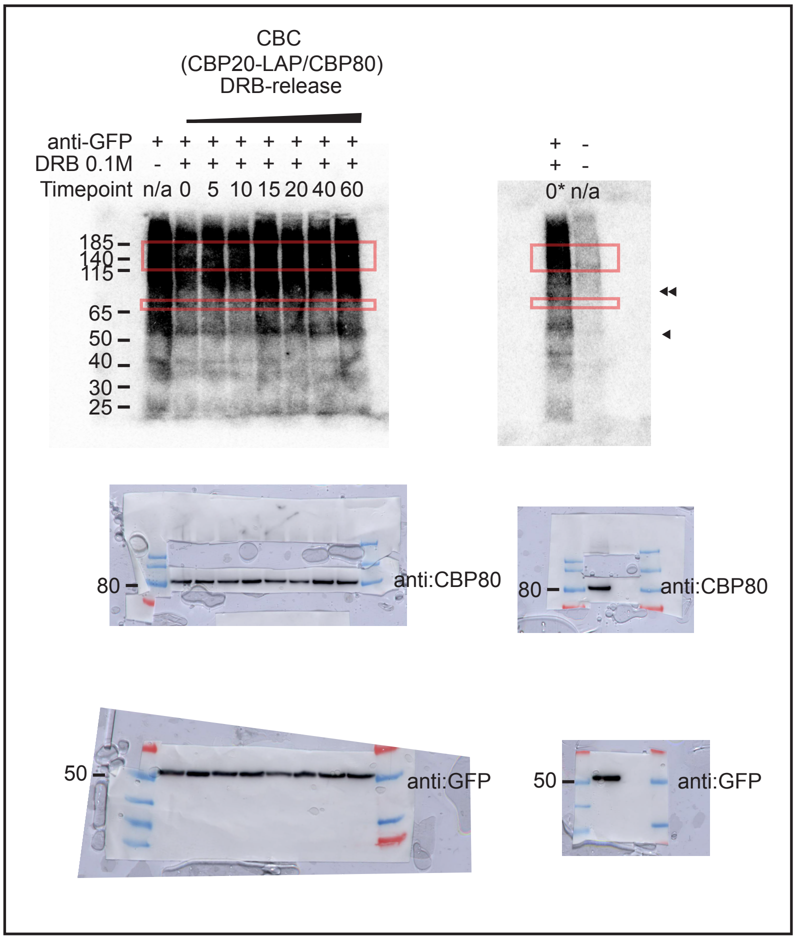

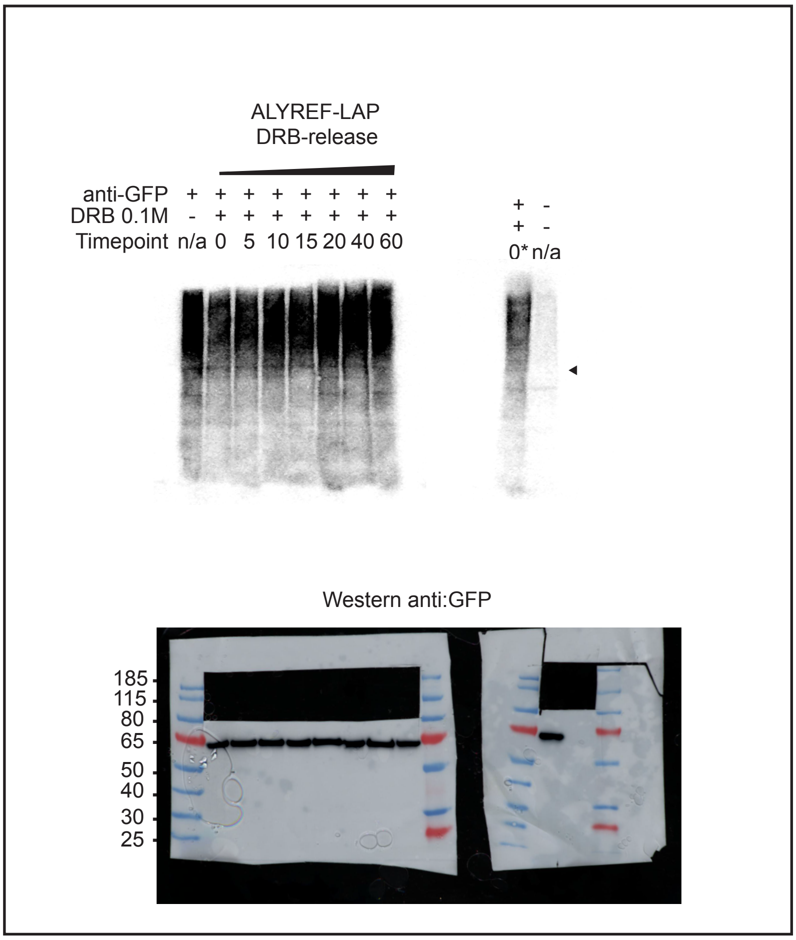

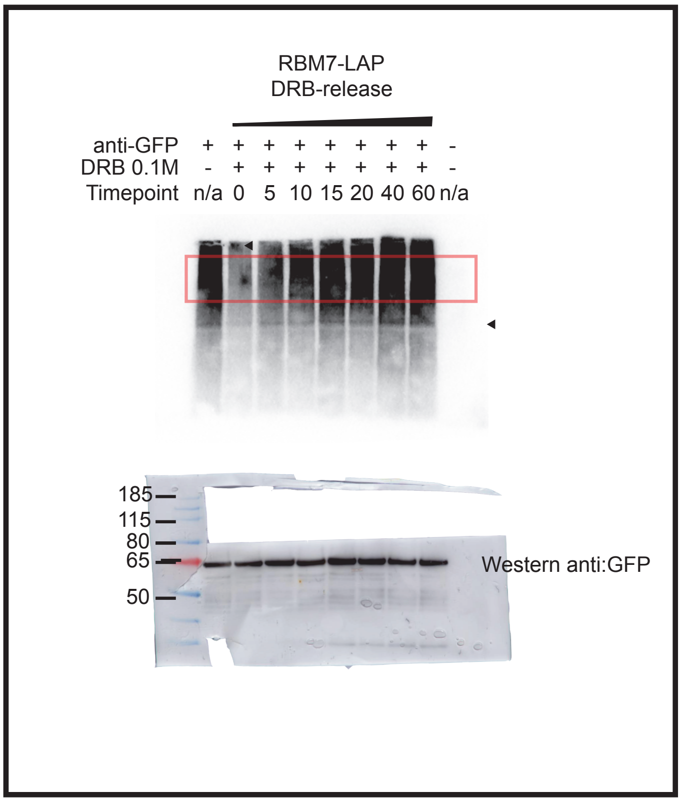

Supplementary Fig. 3e

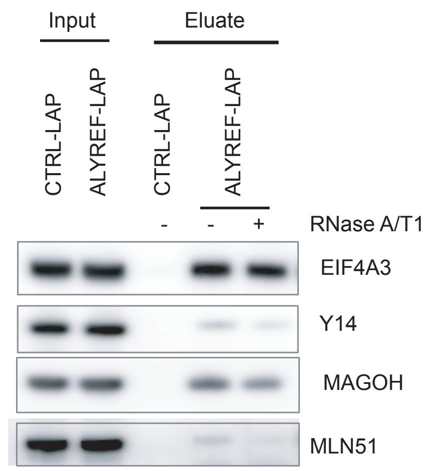

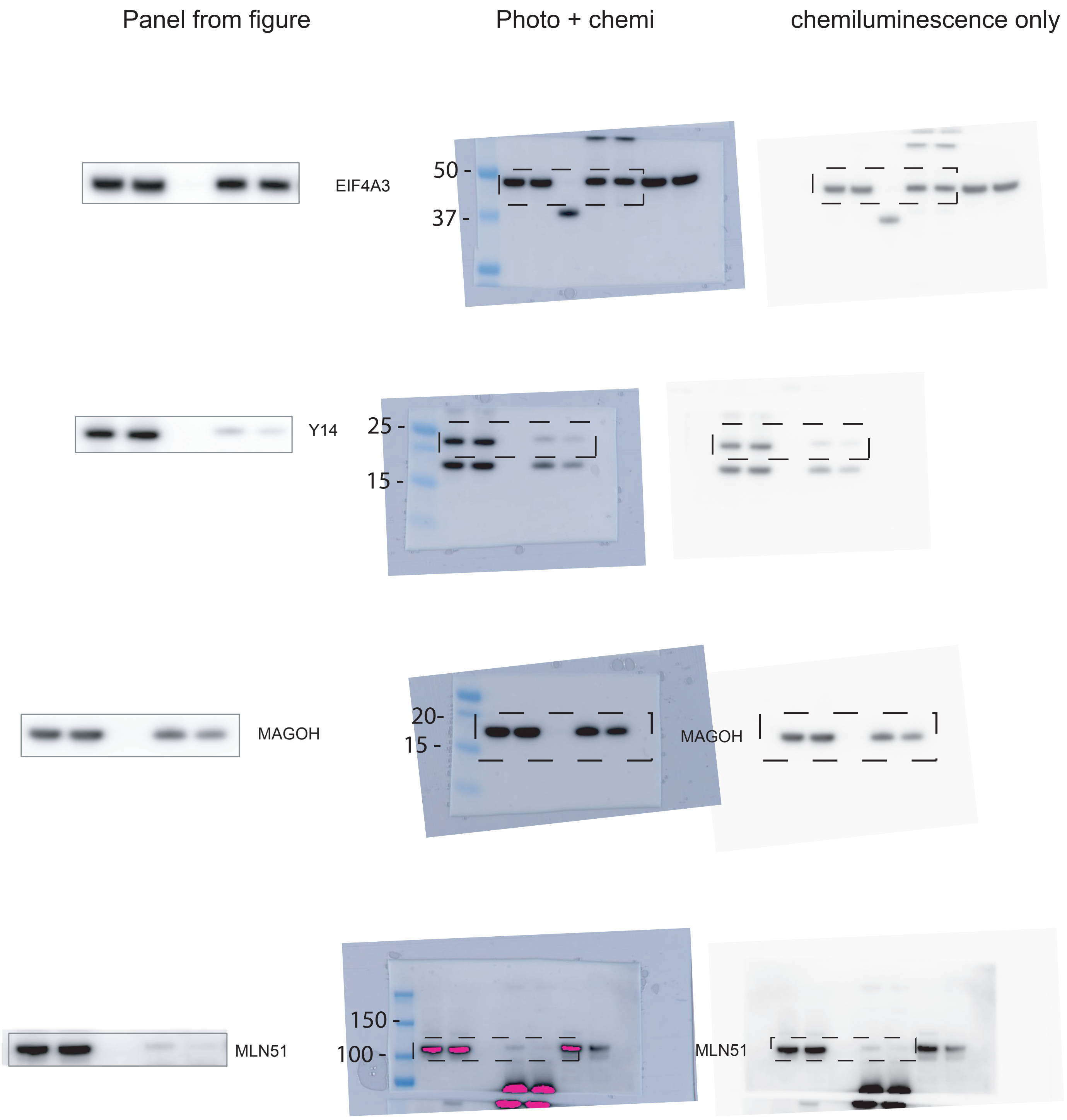

Supplement: Supplementary file 7 — Source Data [file 41467_2023_36345_MOESM7_ESM.zip › Source_data_uncropped_blots_images.pdf]
